# Cognitive, Behavioral, and Situational Influences on Relapse to Smoking After Group Treatment for Tobacco Dependence

**DOI:** 10.3389/fpsyg.2018.02756

**Published:** 2019-01-30

**Authors:** Sara E. Lunden, Jami C. Pittman, Neelam Prashad, Ria Malhotra, Christine E. Sheffer

**Affiliations:** ^1^Department of Community Health and Social Medicine, City University of New York School of Medicine, City College of New York, New York, NY, United States; ^2^Department of Health Behavior, Roswell Park Cancer Institute, Buffalo, NY, United States

**Keywords:** relapse, smoking cessation interventions, cognitive behavior therapy, tobacco dependence treatment, lower socioeconomic status

## Abstract

Socioeconomic disparities in treatment failure rates for evidence-based tobacco dependence treatment are well-established. Adapted cognitive behavioral treatments are extensively tailored to meet the needs of lower socioeconomic status (SES) smokers and dramatically improve early treatment success, but there is little understanding of why treatment failure occurs after a longer period of abstinence than with standard treatment, why early treatment success is not sustained, and why long-term treatment failure rates are no different from standard treatments. We sought to understand the causes of treatment failure from the perspective of diverse participants who relapsed after receiving standard or adapted treatment in a randomized control trial. We used a qualitative approach and a cognitive-behavioral framework to examine themes in responses to a semi-structured post-relapse telephone interview. The primary causes of relapse were familiar (i.e., habit, stress, unanticipated precipitating events). The adapted treatment appeared to improve the management of habits and stress short-term, but did not adequately prepare respondents for unanticipated events. Respondents reported that they would have benefited from continued support. New therapeutic targets might include innovative methods to reduce long-term treatment failure by delivering extended relapse prevention interventions to support early treatment success.

**Trial Registration:** Clinicaltrials.gov NCT02785536.

## Introduction

Cigarette smoking is a leading contributor to socioeconomic health disparities (Mokdad et al., 2004; Jha et al., 2006; American Cancer Society, 2016). The prevalence of smoking among lower socioeconomic status (SES) groups is 2–3 times greater than higher SES groups, leading to significant socioeconomic health disparities (Department of Health and Human Services, 2014; Jamal et al., 2014, 2016). Blacks and African Americans are the largest racial minority group in the United States, comprise 13.2% of the population, but have extraordinarily high poverty rates (25.8%) and are disproportionately represented among lower SES groups (Macartney et al., 2013). Lower SES smokers make more attempts to quit smoking than higher SES smokers; however, lower SES smokers, including a disproportionate number of Black smokers, fail to achieve abstinence more frequently than higher SES smokers (Fagan et al., 2007; Reid et al., 2010; Trinidad et al., 2011; Hiscock et al., 2012; Bosdriesz et al., 2015). Furthermore, smoking-related socioeconomic disparities are increasing despite the success of other tobacco control efforts (Kanjilal et al., 2006; Harper and Lynch, 2007).

While evidence-based multicomponent cognitive-behavioral treatment (CBT) for tobacco dependence is effective for all smokers, there are well-established socioeconomic and racial disparities in treatment outcomes assessed in terms of treatment success (e.g., abstinence or quit rates) and treatment failure (e.g., relapse rates) (Judge et al., 2005; Robles et al., 2008; Hiscock et al., 2012; Sheffer et al., 2013; Varghese et al., 2014). In response to standard, individualized CBT the highest SES smokers are 1.5–2 times more likely to achieve long-term abstinence than the lowest SES smokers (Sheffer et al., 2012a; Varghese et al., 2014). Socioeconomic and racial disparities in treatment failure rates are found for nearly all evidence-based tobacco dependence treatments (Fiore et al., 2008; Robles et al., 2008; Hiscock et al., 2012; Nollen et al., 2017).

Careful, systematic adaptation of standard, individualized CBT for tobacco dependence for lower SES and/or African American or Black smokers improves short-term failure rates for these groups (Sheffer et al., 2017; Webb Hooper et al., 2017). Sheffer et al. (2017) adapted six sessions of standard CBT for lower SES smokers, most of whom identified as Black, using the Barrera and Castro systematic approach for adapting evidence-based behavioral treatments (Barrera and Castro, 2006; Evans et al., 2015). Perspectives from African American and Black smokers were then incorporated using the PEN-3 Model (Airhihenbuwa, 1990, 1992). Webb Hooper et al. (2017) adapted eight sessions of standard CBT for African Americans, most of whom were of lower SES by incorporating deep structure and surface structure topics and elements. Deep structure elements included content framed for specific relevance to African Americans informed by previous research. Surface structure elements included race-matched interventionists, references to the group as a family, session agendas that included traditional African American proverbs or quotations, and using content framed for specific relevance to African Americans and addressing the deep. Both the Sheffer and Webb Hooper studies randomized participants to the adapted or a standard treatment and controlled for treatment contact time.

The adapted treatments in both the Sheffer et al. (2017) and the Webb Hooper et al. (2017) randomized control trials (RCTs) demonstrated significant reductions in short-term treatment failure rates. In the Sheffer et al. (2017) study, significant differences were found for initial 24-h abstinence rates (87.7% vs. 69.0%; *p* = 0.001); the mean number of days to relapse [78.7 (SD 70.4) days vs. 41.9 (SD 62.2) days]; and the median number of days to relapse [66.0 (IQR 7–168) days vs. 8 (IQR 0–49) days]. For the two lowest SES groups, the risk ratios for relapse were one-half to two-thirds of the standard treatment [RR = 0.63 95% CI, 0.45, 0.88, *p* = 0.0013; RR = 0.57 95% CI, 0.18, 0.70, *p* = 0.0024]. In the Webb Hooper et al. (2017) study, the 3-month abstinence rates for the adapted vs. the standard were 36.4% vs. 22.9%, *p* = 0.007. Nonetheless, intermediate relapse rates were remarkably high, the targeted groups were unable to sustain early treatment successes long-term, and no significant differences were found in 6-month abstinence rates in both studies.

The success of the adapted treatments in supporting early abstinence among lower SES smokers is likely due, in part, to tailoring the content of evidence-based interventions to relevant experiences and perspectives. Extensive tailoring common to both the Sheffer et al. (2017) and the Webb Hooper et al. (2017) studies included, but was not limited to, cultural values and beliefs (e.g., spirituality, collectivism, locus of control, etc.), common stressors (discrimination due to race, poverty, or other personal characteristics; economic hardship; financial anxiety; living situations, etc.), and common barriers (menthol, views on medication use, smoking policies in the home) (Evans et al., 2015; Webb Hooper et al., 2017). While the rationale for tailoring supports the early treatment success findings in both studies, there is little understanding of why lower SES smokers relapse precipitously after making such remarkable early success with the adapted treatments.

The process of achieving long-term abstinence from smoking is composed of a variety of behavior changes performed multiple times every day which include establishing new associations (e.g., coping skills) that compete with the original associations (e.g., smoking). The original and the new associations are developed with classical and operant conditioning but despite new learning, the original associations are never completely eliminated (Bouton, 2000), and the contexts in which associations are learned serve as powerful cues. For this reason, recently modified behaviors are inherently unstable and easily swayed by exteroceptive (environmental, background stimuli, etc.) or interoceptive (cognitive, affective, time-related, event-related, etc.) contexts (Bouton, 2000). This conceptualization is consistent with contemporary learning models and Marlatt’s cognitive-behavioral model of relapse (Witkiewitz and Marlatt, 2004). Understanding the factors associated with treatment failure after initial treatment success in the adapted treatment and how these factors might differ or be similar to standard individualized treatment can potentially identify new therapeutic targets to improve long-term treatment failure rates for the adapted treatments.

The aim of this study was to understand the causes of relapse to smoking from the perspective of participants who relapsed after receiving intensive standard or adapted treatment in the Sheffer et al. (2017) RCT. We used a cognitive-behavioral framework and sought to determine the exteroceptive and interoceptive cues associated with treatment failure using a cognitive-behavioral model (i.e., situations, thoughts, and feelings) as well as to examine patterns associated with the adapted and the standard treatment groups. We used a qualitative approach because qualitative methods are particularly valuable when researchers are not able to anticipate the full spectrum of individuals’ experiences or responses (Patton, 2002).

## Materials and Methods

### Participants

Participants (*n* = 227) were recruited into the RCT by word of mouth, fliers placed in the community, and newspaper advertisements. Inclusion criteria included age 18 or older, smoking cigarettes daily, motivated to quit, no regular use of other tobacco products, able to engage in treatment, no contra-indication for use of the nicotine patch, no current use of medications for smoking cessation, drinking < 20 alcoholic drinks per week, negative urine screen for drugs of abuse (e.g., cannabis, cocaine, opiates, methamphetamine, amphetamines, PCP, benzodiazepine, barbiturates, methadone, tricyclic antidepressants, ecstasy, etc.), and attendance of at least one session of treatment. The RCT was conducted between July 2013 and June 2015. Participants who relapsed in the RCT (*n* = 109) were invited by phone to participate in a telephone interview about their relapse experiences in August of 2016. Those who were successfully contacted and agreed to complete the study (*n* = 27) are hereafter identified as respondents. Significance testing compared the characteristics of participants who relapsed and became respondents (*n* = 27) with those who did not (*n* = 82).

### Standard and Adapted Treatments in the Randomized Controlled Trial

#### Standard Treatment

The standard treatment was a well-established, multi-component, manual-driven CBT for tobacco dependence composed of 6 weekly 1-h group sessions and used in numerous programs and studies (Schmitz et al., 1993; Payne et al., 2006; Sheffer et al., 2009, 2012b,a, 2013; Varghese et al., 2014). Standard treatment components included understanding and applying the cue-urge-smoking cycle, developing individualized strategies for managing cues and urges, self-monitoring, guided scheduled rate reduction, goal setting, stress management, problem-solving, conflict management, tobacco refusal training, relapse prevention, enhancing social support, and education about medication and the health effects of tobacco.

#### Adapted Treatment

The adapted treatment was developed from the standard treatment with the goals of addressing treatment outcome disparities and the needs, experiences, and perspectives of diverse lower SES smokers in 6 weekly 1-h group sessions. We used an established framework for adapting evidence-based treatments that included four broad steps:

(1) Information Gathering: Identify modifiable factors that have theoretical and/or empirical support for reducing treatment outcome disparities;
(2) Preliminary Adaptation Design: Incorporate data from Step 1 into a clinical and cultural adaptation;
(3) Preliminary Adaptation Tests: Pilot test the preliminary adaptation from Step 2, obtain community and treatment provider feedback; and
(4) Adaptation Refinement: Incorporate feedback from Step 3 into the final treatment manual (Barrera and Castro, 2006; Lau, 2006). See Evans et al. (2015) for details.

### Approach

We used a cognitive-behavioral conceptual framework to structure the interview, develop interview questions, and interpret participant responses (Steckler et al., 2013). We sought to cultivate an interview context over the telephone in which respondents felt that their opinions were valued and supported open discussion of the reasons why they began smoking again without feeling judged. We made explicit this intention and the encouragement of all perspectives. This approach allowed respondents’ to provide context for their experiences and provided us with the opportunity to understand the full spectrum of participants’ experiences and perceptions within the framework. All interviews were conducted by the same interviewer. The interviewer was blind to respondents’ random assignment in the RCT. Respondents were compensated $10 for their participation. This study was approved by the City University of New York Institutional Review Board (Approval #361627). All participants provided written informed consent for the RCT and continued follow-up. Informed consent was confirmed verbally over the telephone before participants engaged in the interview, which was conducted over the telephone. The datasets used and/or analyzed for this study are available from the corresponding author upon reasonable request.

#### Interview Guide

We began the interview by stating the purpose of the interview and that respondents’ experiences might help to improve treatment in the future. Because we were asking respondents to recall events in the past, we used an anchoring technique similar to that used in a timeline follow-back interview (Sobell and Sobell, 1992) to enhance recall of the specific period of time in which the first relapse occurred after treatment. We provided the dates of first relapse in the RCT and asked about events occurring in their lives at that time, their overall mood, and details about the day they started smoking again such as where they were, time of day, who was with them, and how they obtained cigarettes.

We then asked participants about their thoughts and feelings immediately before smoking the first cigarette of their relapse and then prompted participants to synthesize their thoughts and attribute the relapse to a primary cause. To support saturation of the data, we asked if there was anything else they wanted to tell the interviewer about that time period or why they started smoking again. While closing the interview, we asked if there was anything they found particularly helpful, or would recommend for future programs (see Table 1). Interviews were completed in 10–15 min. Responses were recorded by the interviewer by typing responses into a Qualtrics data collection platform.

**Table 1:** Interview questions.

Situation respondent in time period	I want you to think back to when you were in the study with us. Do you remember what was going on during that time?
	In general, how were you feeling during that time?
Ask for details of situation surrounding relapse	Thinking about the first time you smoked during that period, so on (relapse start date), do you remember where you were?
	What time of day was it?
	What were you doing at that time?
	Who else was with you?
	How did you get that first cigarette?
Specific thoughts, feelings, and reasons for relapse	Immediately before you smoked that first cigarette, what was the thought that went through your head?
	Immediately before you smoked that first cigarette, how were you feeling?
	If you could give one main reason for why you started smoking again at that time, what would it be?
Ensure saturation	Is there anything else you want to tell me about that time period or why you started smoking again?


#### Data Analysis

The data was coded by identifying categories and themes for the responses. The interviewer and two additional research team members independently reviewed the responses, developed coding schemes, and coded the responses. The team members then reviewed the coding schemes together, discussed discrepancies, and agreed on a final coding scheme and a final coding of the responses. The final coding schemes for specific responses were as follows: *Location –* home (inside or outside), work (during or immediately following), other; *Time of day* – morning, afternoon, evening/night; *Activity* – routine, coping with situation; *Other people present* – alone, family/friend(s)/coworker(s)/neighbor(s); *How obtained cigarette* – friends/coworkers/family, purchased it, had it at home; *Thoughts immediately before smoking* – I want that cigarette badly; I don’t care anymore; I shouldn’t be doing this; external factors impacting their situation; *Feelings immediately before smoking* – anxious to smoke; positive affect; negative affect*; Primary reason for relapse* – habit/craving, stressed, event, social influences. All of the above coding options included don’t know/can’t remember.

In addition to the above, two team members independently reviewed each case and assigned a primary reason for relapse from the perspective of the reviewers. The two team members then discussed discrepancies and agreed upon a primary reason. When an agreement could not be reached, a third team member reviewed the case and the three reached a consensus. After the coding was complete, respondents were identified as belonging to either the standard control or the adapted treatment groups and patterns within and between treatment groups were examined.

## Results

Respondents (*n* = 27) were middle aged smokers who were primarily of lower socioeconomic status. The characteristics of the respondents were not significantly different than those of the other participants who relapsed in the RCT with two exceptions: Respondents were slightly older [*M* = 52.0 (*SD* 6.5) vs. *M* = 47.0 *SD* (9.3) years; *F* = 6.5, *p* = 0.01] and generally felt more assured that their basic needs would be met [*M* = 7.7 (*SD* 2.6) vs. *M* = 6.33 (SD 3.3); *F* = 4.12, *p* = 0.04] than the participants in the RCT. Evidence suggests that a sample size of *n* = 27 is more than sufficient to obtain saturation in a qualitative study with fairly narrow objectives (Guest et al., 2006). See Table 2 for respondent characteristics.

**Table 2:** Respondent characteristics (*n* = 27).

Variable	Category or Range	Percent (n) or Mean (*SD*)
Age, y	37–70	52.0 (6.5)
Sex	Male	44.4 (12)
Partnered status	Partnered^1^	18.5 (5)
Race	White or Caucasian	22.2 (6)
	African American or Black	63.0 (17)
	Asian/Pacific islander, American Indian/American Native, Multi-ethic, or more than one race	7.4 (2)
	Other (non-specified)	7.4 (2)
Ethnicity	Hispanic	18.5 (5)
Work status	Full time	18.5 (5)
	Part time	7.4 (2)
	Disabled	14.8 (4)
	Unemployed	55.6 (15)
	Homemaker	3.7 (1)
Socioeconomic status^1^	2–9	4.6 (2.0)
Categories	SES 1	48.1 (13)
	SES 2	40.7 (11)
	SES 3	11.1 (3)
Household income	≤$10,000	51.9 (14)
	$10,000 – $14,999	18.5 (5)
	$15,000 – $24,999	14.8 (4)
	$25,000 – $34,999	0.0 (0)
	$35,000 – $49,999	7.4 (2)
	≥$50,000	7.4 (2)
Education, y	9–16	12.7 (1.7)
Categories	<12 years	14.8 (4)
	12 years	40.7 (11)
	13–14 years	29.6 (8)
	>15 years	14.8 (4)
Health insurance status	Medicaid and/or Medicare	88.9 (24)
	None	3.7 (1)
	Private	7.4 (2)
Deprivation of basic needs	0–10	7.74 (2.6)


*^*1*^A composite index that incorporated household income and educational level (Galobardes et al., 2006a,b). Values assigned to income level (lowest| = |1 to highest| = |6) and educational category (lowest| = |1 to highest| = |4) were combined resulting in a discrete analog SES scale (range| = |2–10). This scale was collapsed into 3 SES levels: SES1 (2–4), SES2 (5–7), and SES3 (8–10) (Sheffer et al., 2012b; Varghese et al., 2014).*

### Situations Associated With Relapse

The majority (70%) of respondents were at home, at work, or on the way home from work when they smoked the first cigarette of their relapse. About half (44%) of respondents initiated relapse in the morning, about one-third (30%) in the afternoon, and 15% in the evening. Two-thirds (67%) of respondents were engaged in routine activities when they initiated relapse, but one-quarter (26%) were actively coping with new or different situations. Examples of routine activities include:

“Just relaxing, watching TV”
“I had just woke up and wanted a cigarette, having coffee and smoking a cigarette”

Examples of attempts to cope with non-routine activities include:

“I had a problem with a supervisor, something in the blueprints that I didn’t read right or he didn’t read right so he tried to bring it to me verbally in a way that I didn’t appreciate. So I just walked away, smoked a cigarette to calm down, went back to re-configurate things”
“I had a bad evening with that person [boyfriend], the relationship was coming to an end. I went to my job and smoked to be consoled.”

About two-thirds (63%) of respondents were alone when they purchased the first cigarette of their relapse; 30% were with family, friends, or coworkers. Nearly one-quarter (22%) obtained their first cigarette from a friend or coworker; 12% already had cigarettes in their homes. Of those that purchased their first cigarette, about half purchased a loosie (i.e., a loose cigarette sold individually).

### Thoughts Associated With Relapse

About one-quarter (26%) of respondents reported thinking about their situation or other external factors prior to relapse.

“When I was going to get out of there [the shelter], because I was there for like 3 years”
“Thinking about the bills I had to pay for that week, thinking about keeping on top of the bills.”

About one-quarter (26%) of respondents reported that they were simply thinking that they wanted a cigarette.

“Smoke it – the thought of the cigarette to my mouth, that’s what I was craving… When I woke up, that was the first thing I wanted, to smoke a cigarette”
“I’m going to enjoy this cigarette.”

About one-fifth (19%) reported that they were thinking that they didn’t care about quitting anymore.

“Screw it, I’ll have a smoke.”
“Why am I punishing myself over not having a cigarette, why am I not sleeping, I’m going to reward myself, there’s no reason to keep doing this over just one cigarette”

### Feelings Associated With Relapse

More than half of respondents (56%) reported negative feelings prior to initiating relapse including feeling depressed, angry, confused, isolated, and stressed.

“Probably feeling down, I need this cigarette to lift my spirit up, it makes me relaxed, start my day off. It’s like a person having a cup of coffee for the job.”
“Mild depression and anxiety. Mostly anxiety because of what I was going through [divorce].”

About one-quarter (22%) reported feeling anxious to smoke.

“Feeling probably anxious to smoke a cigarette, need the nicotine.”
“Anxious, and I was excited that I had a cigarette that I was going to be able to smoke, [it was a] real process, anxiety, relief, I was looking forward to it. I don’t want to sound so serious about it but it was.”

Several respondents (15%) reported positive feelings.

“…calm, wasn’t anxious or anything like that.”
“I was in a good place, I just wanted to smoke a cigarette. Once you’ve been smoking for so long, it’s a habit.”

A few (11%) reported that they felt guilty for intending to smoke.

“I shouldn’t be doing this after going through that whole process, but I did it anyway.”

One respondent reported feeling social pressure from friends to smoke at the same time feeling pressure from his girlfriend not to smoke.

“I told you all I don’t smoke but I was feeling stuffy [stuck-up, snooty]. My girl’s not gonna like it, why you gonna smoke now.”

### Primary Reasons for Relapse

Over one-third (37%) reported that the primary reason they started smoking again was a habit-related craving.

“I still had the habit of smoking, still had the bad habit cause I had been smoking for many years, and it’s hard to stop smoking cigarettes.”
“Addiction, craving a cigarette, [I] really wanted it.”

About one-third (30%) reported stress as the primary reason they started smoking again.

“Probably stress, thinking about the things I have to do, paying bills.”
“Unemployment, [I was] sad because I couldn’t get a job, I was looking for work, couldn’t find a job fast enough. I had high hopes, I was clean, my resume was ready.”

About one-quarter (22%) identified a specific event as the primary reason for initiating relapse.

“When my mom passed away.”
“Hearing something bad, most likely it was a death in the family. It seems like everybody was dying and it was getting more noticeable.”

A few respondents (11%) reported that the primary reason was due to social influences or a lack of social support.

“Probably associations, associations with people who were smoking.”
“Maybe it’s because I get lonely… at the time of the program [the RCT treatment], going to the group and things like that… I would go over there and feel good and like the people that I met. But then I have to cut it off the next day, and then I started smoking again.”

In the majority of cases (85%), the respondents’ primary reason for relapse matched the reviewers’ conclusions; however, the reviewers classified the primary reason differently than the respondents in 4 of the 27 cases. In one of these cases, the respondent reported the primary reason was stress:

“Stressing, it makes me feel more comfortable. It’s an old habit, something I was used to, it was just so comfortable.”

The reviewers, however, noted that respondent discussed being outside with friends and neighbors when he started smoking:

“Out with a couple of friends and they were smoking, normally I smoke. They offered cigarettes which makes it hard.”

When asked how he was feeling before he started smoking the first cigarette of his relapse, the respondent said,

“Odd man out.”

In reviewing this case, the reviewers decided that while stress played a role, a precipitating social event played a significant role in initiating the relapse.

In another instance, the respondent concluded the primary reason was:

“Stress and alcohol. I didn’t have to think about it. And habit, smoking cigarettes is not just the nicotine, but kind of like when you drink, you want to smoke; the two go together.”

The reviewers, however, noted that the relapse was initiated after a

“Fight with my supervisor, so I went outside to calm down.”

While stress clearly played a role, there appeared to be a precipitating event as well. We used the reviewers’ determinations to quantify the primary reasons for relapse.

About one-third (37%) of relapses were attributed to craving related to habit. Responses included:

“Just a bad habit.”
  “…just not thinking before I had it.”

About one-third attributed the primary reason to stress. Responses included:

“Stress, in general, from the living situation.”
“I would say stress and a little bit anxiety.”

About one-quarter attributed the reason to a precipitating event such as,

“Started when my mom passed away.”
“Concerned about the cancer…”

The primary reason for relapse, for a few, was related to social influences,

“Probably associations, association with people who were smoking”
“…being with some people…”

### Patterns Associated With Standard and Adapted Treatments

The respondents were distributed similarly between the two conditions; 41% received the standard treatment and 59% received the adapted treatment. Nearly half (46%) of the respondents who received standard treatment reported habit as the primary reason for relapse, while 31% of respondents who received adapted treatment reported habit; 36% of respondents who received standard versus 25% of respondents who received adapted attributed stress as the primary reason. Only 9% (*n* = 1) of respondents who received standard versus 31% of those who received adapted reported a precipitating event. Only 9% of the respondents who received standard versus 12.5% who received adapted attributed the primary reason to social influences.

### Respondent Comments About the Treatment

Many respondents offered comments and suggestions about the treatment. A common theme from nearly all respondents who offered advice was that the assistance should have been extended. Responses included:

“Yeah, you could, you know, follow up more, maybe give us another set of you know patches or whatever. Have more meetings, sitting with people that are in the same boat as you are, easy to follow you know.”
“…just stay in touch more for the participants, see how they’re doing…”
“…maybe longer sessions, maybe 15–20 min longer.”
“Most beneficial was the actual group itself, I did the patch, I don’t know maybe, if it had been longer, yeah, it would have been more helpful if it had been longer, for me I felt like the supportive group was one of the most beneficial because I almost had to like show up and be like, you had to, for me it would be embarrassing if I had smoked a cigarette, but being held accountable, it, it would have been better if it was longer, maybe a year, 9 months.”
“Outside support, more meeting, should have a smokers group, voluntary, not because they’re paying you, but because you really want to quit smoking, sort of like an AA type of thing but a smoking one.”
“…Maybe, just talking about um, skills and checks for delayed gratification, learning that a little bit more, hammering that into your head, what to do in the 5 min where the crave left, not just distractions, but really sitting down and building muscles for delayed gratification.”
“…some kind of back up support maybe once a month that a person could come back, say a couple of months afterward so that people could come back and check in.”

One respondent mentioned that assessing carbon monoxide (CO) level in exhaled breath was helpful:

“…if you were able to give people those blower things [CO monitors], that really helped because I know on the days where I would lie, I definitely would not smoke about 8 h before, because I didn’t want a bad read, if they’re affordable, would be better; before smoking would be able to see. That would be a good carrot for me.”

## Discussion

The strengths of this qualitative study include an in-depth examination of details using a conceptual framework strongly linked with the treatment approach as well as a relatively large number of subjects supporting thorough saturation of the data. The findings suggest that after an intensive treatment for tobacco dependence, participants tended to relapse in their usual environments, during the morning or afternoon, and while engaged in routine activities, although some were coping with new or different situations. Many were alone, but some were with family, friends, or co-workers, and many took advantage of the availability of loose cigarettes for sale in their neighborhoods. Many were also experiencing negative feelings when they relapsed, including feelings of depression and anxiety, while a few reported that the relapse was precipitated by positive feelings. The primary causes of relapse to smoking included strong cravings associated with habit, managing stress, coping with unexpected or stressful events, and to some extent managing social pressure to smoke. Most participants who made recommendations about improving the treatment indicated that they would have benefited from continued or additional support.

Qualitative findings often suggest patterns and differences to be explored with future hypothesis testing. Participants who received standard treatment relapsed, on average, 37 days earlier than participants in the adapted treatment. Respondents who received standard treatment were more likely to relapse earlier in response to habit or stress which makes sense given that respondents in the adapted treatment had more days of successful experience than those in the standard treatment and the adapted treatment included a stronger emphasis on stress management than the standard treatment. Respondents who received adapted treatment, however, appeared to relapse more frequently from external, unanticipated precipitating events. Social influences appeared to affect relapse among respondents in both conditions fairly equally. These findings suggest that treatment failure is associated with inadequate preparation for the management of cued behaviors and inadequate stress management preparation in the standard treatment and to a lesser extent in the adapted treatment. For the adapted treatment, among those who managed these factors adequately, treatment failure was associated with inadequate preparation for managing unexpected events without smoking.

These findings suggest therapeutic targets to enhance relapse prevention for lower SES groups. Respondents in the adapted treatment were able to implement new associations, new coping skills, and new learning during early and short-term abstinence (up to about 3 months post-quit), but appeared unable to sustain new learning longer term in context. Contemporary learning models indicate that providing retrieval cues, situating new learning in relevant contexts, and varying the contexts in which new learning takes place can improve the long-term maintenance of new associations (Bouton, 2000). Interventions that extend the learning established in treatment by emphasizing rehearsal of new associations in context and providing long-term exposure to retrieval cues might reduce long-term treatment failure. Applying these strategies to manage habit-related cravings, stress, unexpected stressful situations, negative affect, and social influences might reduce the precipitous rate at which lower SES smokers relapse after attaining considerable success in the adapted treatments. These conclusions are supported by respondents comments. Many respondents reported that they would have benefited from continued support. Innovative methods to reduce treatment failure might include delivering this support with methods and mediums that do not require face-to-face interactions. Extended relapse prevention interventions would likely benefit from the tailored approaches utilized in the adapted treatment in the Webb Hooper et al. (2017) and the Sheffer et al. (2017) studies.

Finally, specific cue-related or self-regulation strategies for avoiding the purchase of loose cigarettes might enhance relapse prevention interventions as well. At least 50% of the respondents indicated that the first cigarette of their relapse was purchased as a “loosie,” a cigarette sold individually, usually for one dollar or less. Selling loosies is illegal and retailers who sell cigarettes are subject to compliance inspections under the Family Smoking Prevention and Tobacco Control Act of 2009 (U.S. Food and Drug Administration, 2009). Loosies are more commonly available in lower income neighborhoods from corner stores, bodegas, or from individuals (Smith et al., 2007; Stillman et al., 2007). Loosies enable smokers to reduce the immediate cost of smoking, even though smoking loosies might in the long-term cost more per cigarette than purchasing a pack of cigarettes. Our findings suggest that the availability of loose cigarettes enhances treatment failure among newly quit individuals. The availability of loosies diminishes the public health impact of increasing cigarettes taxes. Residents of the neighborhoods where loosies are available are thus not benefiting equally from this powerful tobacco control strategy, representing yet another tobacco-related socioeconomic disparity.

### Limitations

This qualitative study used non-probabilistic sampling and thus the findings might not generalize to larger populations. Despite our attempts to minimize recall bias by using anchoring techniques, participants’ reports remain subject to recall bias. In addition, while we asked for participants by name and discussed data we had collected previously such as the timing of the first relapse, because the interview was conducted over the telephone the identity of participants could not be verified. Nonetheless, the minimal compensation, a $10 check, did not support coercion, and there was little reason for an individual to impersonate a participant. The low level of compensation might also have dis-incentivized higher SES participants.

Future research might replicate these findings in representative samples as well as examine enhanced relapse prevention methods as new therapeutic targets for lower SES groups. Therapeutic targets to enhance relapse prevention among lower SES groups might include augmenting treatment by rehearsing new learning in relevant contexts, varying the contexts in which new learning takes place, and providing long-term exposure to retrieval cues to support the management of habit-related cravings, chronic and acute stress, negative affect, and the availability of loose cigarettes.

## Ethics Statement

This study was carried out in accordance with the recommendations of Code of Federal Regulations Title 45 Public Welfare Department of Health and Human Services Part 46 Protection of Human Subjects. The protocol was approved by the Institutional Review Board of the City University of New York. All subjects gave informed consent in accordance with the Declaration of Helsinki.

## Author Contributions

CS developed the concept and led the team who conducted this project. JP developed the database and contributed to the development of the study design. SL refined the design, conducted the study, and led the development of the coding protocols. NP and RM contributed to coding the data and the interpretation of the results. CS and SL developed the manuscript. All authors were involved with revising the manuscript and have given final approval for publication.

## Conflict of Interest Statement

The authors declare that the research was conducted in the absence of any commercial or financial relationships that could be construed as a potential conflict of interest.
